# Two-Dimensional Ferroelectric Materials: From Prediction to Applications

**DOI:** 10.3390/nano15020109

**Published:** 2025-01-12

**Authors:** Shujuan Jiang, Yongwei Wang, Guangping Zheng

**Affiliations:** 1 Collaborative Innovation Center of Steel Technology, University of Science and Technology Beijing, Beijing 100083, China; shujuan.jiang@connect.polyu.hk; 2 Department of Mechanical Engineering, Hong Kong Polytechnic University, Hong Kong 999077, China

**Keywords:** ferroelectrics, 2D materials, slidetronics, spintronics, first-principles calculation

## Abstract

Ferroelectric materials hold immense potential for diverse applications in sensors, actuators, memory storage, and microelectronics. The discovery of two-dimensional (2D) ferroelectrics, particularly ultrathin compounds with stable crystal structure and room-temperature ferroelectricity, has led to significant advancements in the field. However, challenges such as depolarization effects, low Curie temperature, and high energy barriers for polarization reversal remain in the development of 2D ferroelectrics with high performance. In this review, recent progress in the discovery and design of 2D ferroelectric materials is discussed, focusing on their properties, underlying mechanisms, and applications. Based on the work discussed in this review, we look ahead to theoretical prediction for 2D ferroelectric materials and their potential applications, such as the application in nonlinear optics. The progress in theoretical and experimental research could lead to the discovery and design of next-generation nanoelectronic and optoelectronic devices, facilitating the applications of 2D ferroelectric materials in emerging advanced technologies.

## 1. Introduction

Ferroelectric (FE) materials possess spontaneous polarization below a critical temperature (T_c_, the Curie temperature), which can be reversed by applied electric fields. This fascinating property, arising from broken spatial symmetry and a shift in negative and positive charge centers, enables their use in a variety of functional devices, including sensors, actuators, memory for information storage, and field effect transistors [1,2,3]. Additionally, the switching of electric dipoles often induces field-dependent changes in volume, giving rise to piezoelectricity, while the temperature-dependent polarization of FE materials grants them with pyroelectric properties. Because of these unique properties, the studies on FE materials and their industrial applications are of great interest.

The discovery of ferroelectricity in barium titanate (BaTiO_3_) in 1946 marks an important milestone for the practical application of FE materials [4]. Since then, bulk perovskite oxides, as described by the chemical formula ABO_3_, have become the largest family of FE materials owning to advances in fabrication and preparation technologies [5,6,7]. Recently, two-dimensional (2D) ferroelectricity has been discovered in various ultrathin compounds with various types of 2D crystal structures, driven by advances in theoretical computation and modeling [8,9,10]. These studies play an important role in resolving the long-standing issues of low-dimensional ferroelectrics. Generally, as the thickness of ferroelectric films approaches a critical value, typically several nano-meters, their ferroelectricity encounters challenges, such as depolarization effects and structural instabilities caused by dangling bonds and surface reconstructions [11]. In combination with lattice mismatch induced by substrates and the complex fabrication processes, the development of high-quality ultra-thin ferroelectric films suitable for multifunctional and microelectronic applications remains limited.

The successful mechanical exfoliation of graphene by Novoselov et al. in 2004 ushered in a new era of 2D van der Waals (vdW) materials. Emerging 2D families with novel optical, electrical, and mechanical properties, such as transition metal dichalcogenides (TMDs) [12,13], hexagonal boron nitride [14], and MXenes [15], have become the research hot spots in the past two decades. In particular, the reduction of dimensions may give rise to the broken symmetry of the materials, leading to their intrinsic ferroelectricity with stable polarization. The strong intralayer bonds and weak interlayer coupling provide a pathway to realize sustainable ferroelectricity in 2D materials with free surfaces, leading to a new family of FE materials, i.e., free-standing 2D ferroelectric materials. So far, over one hundred 2D FE materials have been theoretically predicted, including 1T-MoS_2_, In_2_Se_3_, CuInP_2_S_6_, and MX (M = Ge, Sn; X = S, Se). The strong polarization and room-temperature stability of these 2D materials promote the prosperous evolution of nanoelectronics. Nonetheless, due to the weak FE signals, only a few FE nanosheets have been confirmed in experiments, such as α-In_2_Se_3_ [16], CuInP_2_S_6_ [17], SnTe [18], BA_2_PbC_l4_ [19], *d*1T-MoTe_2_ [20], and WTe_2_ [21]. Besides their limited availability, designing and preparing promising 2D ferroelectrics with strong polarization and moderate Curie temperature remains challenging, and there exist the difficulties in meeting the demands for miniaturization and multifunctionality in their practical applications. It is worth noting that, despite being in their infancy, the emerging 2D ferroelectrics offer a unique platform for the implementation of nanotechnology.

To explore 2D FE materials with desired properties, two common approaches are pursued, as follows: first, to discover novel polar 2D materials with intrinsic ferroelectricity; second, to induce ferroelectricity in nonpolar materials through strain engineering or modulating stacking orders of the monolayers. In this review, major 2D FE materials are introduced, providing an overview of current advancements of these novel ferroelectrics. Furthermore, the underlying mechanisms of 2D ferroelectricity and potential applications of 2D FE materials are discussed, and the pathways for developing novel 2D FE materials are suggested.

## 2. Computational Methods for the Design of 2D FE Materials

Among the theoretical analysis approaches, the first-principles calculations based on density functional theory (DFT) have been widely used to investigate the physical and chemical properties of macroscopic structures and to reveal the microscopic mechanisms. This method solves the Schrodinger equation without using adjustable parameters, resulting in an improved accuracy of analysis on the physical and chemical properties. In the first-principles calculation, the stable polar states of 2D FE systems could be determined by searching for the local minimum of free energy. It should be noted that some 2D materials have more than one stable phase. Meanwhile, unstable 2D structures can be excluded by judging their phonon spectrum with the negative frequency, which can be obtained from the calculation using PHONOPY code [22]. The FE strength is determined by the magnitude of spontaneous polarization, which is defined as the density of electric dipole moment and can be quantitatively computed using Berry phase calculations, based on modern polarization theory [23,24]. Furthermore, the existence of switchable polarization with an appropriate energy barrier under applied electric fields is an indication of ferroelectricity in 2D FE materials. In general, double- or quadruple-well potential models and the climbing image nudged elastic band method [25] could aid in the discovery of transition states and the determination of the lowest energy path for the reversal of polarization with a polar phase. Despite the complex underlying mechanisms of ferroelectricity, the soft-mode theory is a viable option for revealing its origin. The soft mode occurring in the paraelectric (PE) phonon spectrum corresponds to possible atomic movement that can lead to stable or competitive polar states. Supplemented by the analysis of its electronic or mechanical properties, a novel 2D FE material could be determined by the aforementioned first-principles calculations.

## 3. Major Classes of 2D FE Materials

### 3.1. Copper Indium Thiophosphate

**Figure 1:**
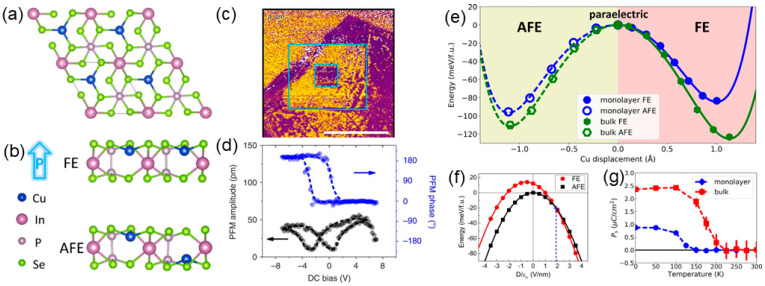
(**a**) Top view and (**b**) side view of the CuInP_2_S_6_ monolayer [29]. (**c**) The PFM phase images for the 4 nm-thick CIPS flakes with reversed DC bias [28]. (**d**) The corresponding PFM amplitude (black) and phase (blue) hysteresis loops during the switching process for the 4 nm-thick CIPS flakes [28]. (**e**) Energetics of the CuInP_2_Se_6_ monolayer and bulk sample as determined by first-principles nudged elastic band (NEB) calculations, revealing the FE(AFE)–to–paraelectric phase transitions [29]. (**f**) Energy variation with aspect to the applied electric field *D*/*ε*_0_ [29]. (**g**) Temperature-dependent zero-field spontaneous polarization *P*_*s*_ in monolayer and bulk CuInP_2_Se_6_ [29]. Reprinted with permission from [29], 2017, American Physical Society; and [28], 2016, Springer Nature.

Copper indium thiophosphate CuInP_2_S_6_ (CIPS) monolayer and its many analogs, referred to as ABP_2_X_6_, where A/B is a transition metal and X is chalcogenide including S, Se, and Te, form a class of 2D FE materials. In the 2D atomic structure of ABP_2_X_6_, X atoms locate at the top and bottom layers, and P-P bonds are symmetric, while A and B atoms fill the octahedral clusters alternatively, with different distances away from the center atomic layer along the z-direction, as shown in Figure 1a,b. In 1994, a first-order PE-to-FE phase transition at 315 K in bulk CIPS was discovered in experiments, which was the first observation of ferroelectricity in lamellar materials [26]. Three years later, according to the X-ray and neutron diffraction investigations, the ferroelectricity was identified as the result of the thermal hopping of Cu (I) atoms in the PE phase [27]. In 2015, the stable FE polarization was firstly measured in layered CIPS using piezoresponse force microscopy (PFM) [17], and the domain structures, switchable polarization, and hysteresis loops provided direct evidences. When the thickness is less than 50 nm, the ferroelectricity disappears due to the depolarization field. However, Liu et al. conducted the local switching tests and discovered that CuInP_2_S_6_ ~4 nm in thickness could maintain the feature of FE switching [28]. Under a DC bias applied between PFM tips and the silicon substrate, an obvious reversal of phase contrast occurred after the DC bias was reversed, as shown in Figure 1c. The typical butterfly loops of PFM amplitude signal and phase switching shown in Figure 1d also confirmed the existence of ferroelectricity in CIPS. Its room-temperature stability further verified that the PE-to-FE phase transition temperature is about 320 K, opening up new possibilities for developing nanoscale devices based on 2D FE materials [28].

Unlike its FE bulk counterparts, the CuInP_2_Se_6_ monolayer was reported to exhibit an antiferroelectric (AFE) ground state, with a six-layer critical thickness under the open-circuit boundary conditions [29]. Because of the small energy difference and large energy barrier for the AFE and FE states, the FE state useful for device applications could be practically stable. Experimental studies revealed that there was a coexistence of FE and AFE states in CuInP_2_Se_6_. Although the AFE states have lower energy than the FE states, CuInP_2_Se_6_ is stabilized at its FE state since the energy barrier between AFE and FE states is huge, as shown in Figure 1e–g [29]. In addition, the theoretical studies revealed that the ground state of AgBiP_2_Se_6_ was also not purely FE because the out-of-plane (vertical) ferroelectricity could be originated from the compensated FE orders, reducing the depolarization field and stabilizing the ferroelectricity of AgBiP_2_Se_6_ with a thickness up to 6 Å [30]. Besides their superior ferroelectricity for nanoelectronic applications, these 2D FE materials exhibit outstanding visible-light absorption and suitable band alignment, which are promising for photocatalysis applications.

The ferroelectricity of 2D FE systems with stable AFE/PE states can be further manipulated for their practical applications. The manipulation of the ferroelectricity is implemented through the application of mechanical strains and/or electric fields, which have been proved to be effective [31]. Yu et al. tuned the AFE ABP_2_X_6_ to possess FE ground states through interfacing it with graphene, MoS_2_, h-BN, MoTe_2_ monolayers, and polar MgO (111) surfaces, facilitating their practical device applications. A reasonable explanation for this mechanism is that there is coupling between charge polarization in monolayers and the local dipole, as well as electronic polarization in the substrates [32].

Being a class of 2D multiferroic semiconductors that have both ferroelectricity and ferromagnetism, ABP_2_S_6_ monolayers provide a platform for realizing nanoscale switches and memory devices [33]. The spontaneous motion of A/B atoms away from the centrosymmetric plane gives rise to the electric dipole moment, which can be modulated through applying an external electric field. For monolayer and few-layer CuCrP_2_S_6_, FE and FM states were observed rather than AFE and AFM states in their bulk counterparts. The spins, electric dipoles, and free-energy valleys of 2D CuCrP_2_S_6_ are coupled with each other, as demonstrated by theoretical calculations and experiments [34]. It was reported that two modes of polarization (Γ_2_^−^ and Γ_1_^+^) in ABP_2_S_6_ could lead to ferroelectricity and anti-ferroelectricity in the system [35]. The antipolar mode Γ_1_^+^ related to AFE distortion leads to the FE distortion unexpectedly and influences the magnetic anisotropic axis, resulting in FE and FM states in 2D ABP_2_S_6_.

**Figure 2:**
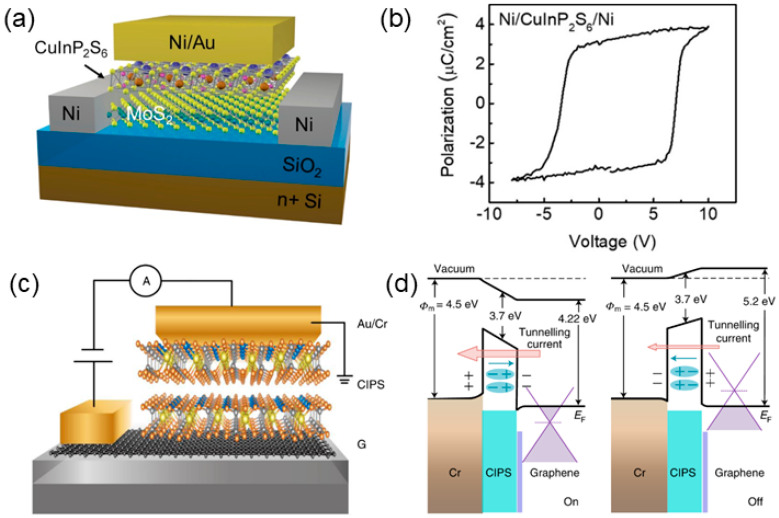
(**a**) Schematic diagram of MoS_2_/CIPS 2D FE-FETs [36]. (**b**) Polarization–voltage loop for FE capacitor at 290 K [36]. (**c**) Schematic of Cr/CIPS/graphene FTJ on the SiO_2_/Si substrate [37]. (**d**) Band diagrams for the on- and off-states of the vdW FTJ operation [37]. The built-in polarization fields are indicated by cyan arrows. Reprinted with permission from [36], 2018, American Chemical Society; and [37], 2020, Springer Nature.

2D FE field-effect transistors (FETs) are the representative devices that utilize FE switching to control the conducting channel and achieve the on/off states. The integration of 2D FE materials with 2D semiconductors results in vdW heterostructures, which could effectively avoid interface problems and improve the performance of FET. Such strategy was implemented by constructing a CIPS monolayer on the top of MoS_2_, as shown in Figure 2a [36]. Without dangling bonds, the as-constructed heterostructure offers a semiconductor/insulator interface. The polarization versus voltage curves exhibit a well-defined hysteresis loop, indicating the ferroelectricity of the heterostructure, as shown in Figure 2b [36]. Importantly, the measured on/off ratio can reach 10^4^ through tuning the capacitance matching, which can be modulated by the back-gate bias of the MoS_2_ transistor. As an FE switchable diode, CIPS/Si heterostructure exhibits good memory behavior, with an on/off ratio of about 100 [28]. Moreover, the FE tunnel junction (FTJ) of Cr/CIPS/graphene layered heterostructures also show high tunneling electroresistance (TER, >10^7^), where CIPS can modulate the FE barrier [37]. Figure 2c shows the schematic of the FTJ. Such giant TER could be explained by the influence of FE polarization on the Fermi level of graphene. For the on-state, n-type graphene leads to the up-shifted Fermi level, while during the off-state, the switched polarization of CIPS induces the down-shifted Fermi level, as shown in Figure 2d.

Besides their applications in nanoelectronic devices, ABP_2_X_6_ systems are promising for water-splitting applications. The AgBiP_2_S_6_ monolayer was reported to have excellent photocatalytic properties, with the photocatalytic efficiency adjusted by FE-to-PE phase transition [38]. The FE phase is beneficial for water oxidation due to a higher concentration of photogenerated holes, while the PE phase is favorable for hydrogen reduction, resulting from a higher concentration of photogenerated electrons. The solar-to-hydrogen energy conversion efficiency reaches 10.04% for 2D ABP_2_X_6_ with the FE phase, providing a new avenue for photocatalysis via FE switching. In particular, 2D ABP_2_S_6_ is predicted to screen the FE monolayers exhibiting out-of-plane electric polarization [39]. Remarkably, CIPS possesses the largest vertical polarization (0.59 μC/cm^2^) on account of the larger off-center shift of metal atoms, and that of CuBiP_2_S_6_ is relatively smaller (0.35 μC/cm^2^). Their Cu-S bonds are also responsible for the high phase-transition temperatures. The polarization can persist in the vdW heterostructures, whose band alignment could facilitate photocatalytic water splitting.

With the emergence of neuromorphic computing, the artificial synapse is a critical block of the circuits. Those fully made of ReS_2_/hBN/CIPS vdW materials were reported to exhibit an on/off ratio exceeding 10^7^, realizing the optical controlling of the polarization and thereby demonstrating a fresh avenue for future research on FE heterostructures [40].

### 3.2. Transition Metal Dichalcogenides (TMDs)

**Figure 3:**
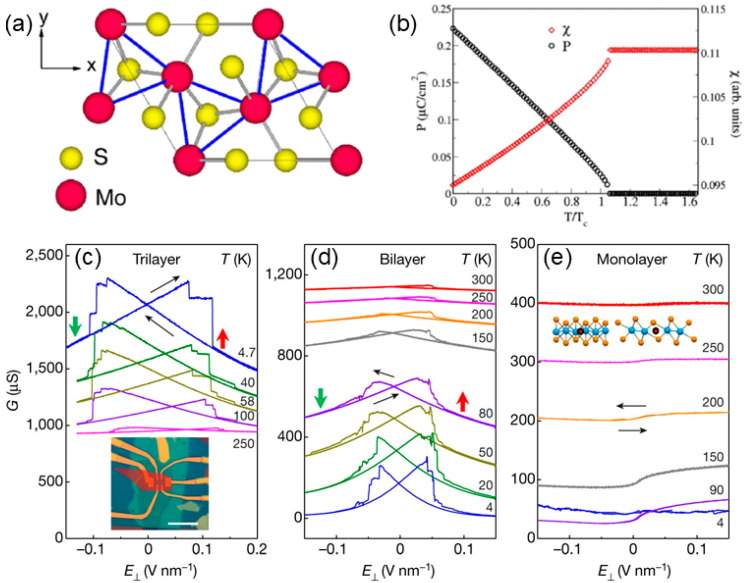
(**a**) Trimerization of Mo atoms in the 1T crystal structure with a √3×√3 unit cell [41]. (**b**) Polarization (P) and dielectric susceptibility (χ) as a function of temperature derived from Landau theory [41]. (**c**–**e**) Conductance *G* of undoped trilayer, bilayer, and monolayer device as *E*_⊥_ is swept up and down (black arrows) [21]; The polarization is represented by a green or red arrow in (**c**); The location of a centre of symmetry is represented by a red dot in (**e**). Reprinted with permission from [41], 2014, American Chemical Society; and [21], 2018, Springer Nature.

Ten years after the discovery of graphene, the distorted 1T-MoS_2_ monolayer was reported to be FE with an out-of-plane polarization of 0.28 μC/cm^2^, as studied by DFT calculations and analyzed by Landau theory [41]. Such unexpected vertical ferroelectricity was the result of nonlinearly coupled structure distortion, as shown in Figure 3a. Because of the degeneracy of the Fermi surface and strong electron–phonon coupling, the ferroelectricity mainly resulting from the Mo-induced spontaneous symmetry breaking could be switchable with a gate field. Considering the activation energy (0.23 eV/f.u.), as shown in Figure 3b, the above-room-temperature transition temperature was estimated by mean-field theory. Two-dimensional MoSe_2_, WS_2_, and WSe_2_ were also confirmed to possess ferroelectricity, offering tunability in the design of devices based on 2D chalcogenides. In addition, MX_2_ (M = Mo, W; X = S, Se, Te) semiconductors with d^2^ metal ions were predicted to show reversible polarization [42]. The $\Gamma_{2}^{-}$ mode of additional distortion was responsible for the inversion-symmetry breaking that resulted in the FE state, stimulating further experimental investigations of relevant phenomena.

In 2018, the first experimental verification of ferroelectricity in this family was carried out in semimetal WTe_2_ with two or three layers [21]. The stacking structure was thin enough to respond to an applied electric field, exhibiting spontaneous polarization. The switching bistability, which was an essential characteristic of the FE state, was observed in the trilayer and bilayer with the applied electric field, while no stability could be observed in the monolayer, as shown in Figure 3c–e. Importantly, this stable ferroelectricity could be well maintained until 350 K, suggesting its promising switching capability in devices. Liu et al. clarified the working mechanisms and predicted its potential applications in spintronics. It was indicated that the polarization directions further controlled the spin texture of the WTe_2_ bilayer, which could facilitate the design of spin field-effect transistor [43]. Furthermore, the distorted 1T-MoTe_2_ with strong out-of-plane ferroelectricity was exploited by Yuan et al. A large on/off resistance ratio (about 1000) was achieved in the MoTe_2_-based FE tunneling junction, showing great potential in atomic-scale logic devices [20].

**Figure 4:**
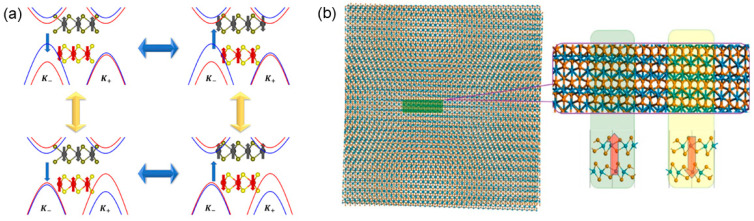
(**a**) Multi-state (green and red arrows indicate the polarization of states) control of 2D multiferroic bilayer VS_2_ [45]. (**b**) FE domains formed by Moiré patterns upon a small angle twist of a VTe_2_ bilayer [47]. Reprinted with permission from [45], 2020, American Chemical Society; and [47], 2018, American Chemical Society.

The discovery of ferroelectricity in atom-thick monolayers is still limited. In 2017, Li et al. revealed the existence of vertical ferroelectricity in a series of vdW bilayers by the first-principles calculations [44]. The ferroelectricity can be induced in nonferroelectric semiconducting systems by stacking bilayers with broken inversion symmetry. In these systems, the polarization origins from the vertical charge transfer between uncompensated interlayers due to in-plane translation. Resulting from the interlayer sliding, the electrical polarization is switchable, and the systems could be sliding FE. Among them, bilayer MoS_2_ shows a vertical polarization of 0.97 × 10^−12^ C/m, stemming from the interlayer voltage induced by interlayer charge transfer between Mo-S atoms. Similarly, bilayer ferromagnetic VS_2_ could become multiferroics with switchable magnetization upon FE switching, rendering it with efficient reading and writing for high-density data storage. Additionally, the out-of-plane polarization in bilayer VS_2_ with R-type stacking can be also reversed upon interlayer sliding, which results in the control of magnetism [45]. As shown in Figure 4a, four types of FE antiferromagnetic structures can be achieved by external magnetic fields and electric fields, which are promising for multi-states storage. The calculated polarization value is 2.018 × 10^−3^ C/m^2^, larger than the experimental results reported previously. To understand the coupling between different degrees of freedom in such systems, Ma et al. developed a model to unveil the relationship between the polarization induced by interlayer sliding and the energy difference between K+ and K− valleys of energy landscapes [46]. The model provides an alternative approach in quantitatively studying the polarization in sliding FE systems.

Compared with traditional ferroelectrics, it is convenient to form and tune a Moiré superlattice pattern of FE domains with opposite direction. For example, the vertical polarization was predicted in the WT_2_ bilayer, and the Moiré superlattice could be formed by a small angle twist of the bilayer [47]. Depending on the angle and position, the domain size and local polarization can be modulated, as shown in Figure 4b. Besides the TMD bilayer, room-temperature ferroelectricity was also realized in TMD multilayers. For IT’-ReS_2_ with layer number N ≥ 2, the vertical ferroelectricity can be obtained [48]. As N increases, the polarization value and the transition barrier both increase gradually. With the progress in materials synthesis and experimental measurement, the sliding FE systems have been verified with the TMD bilayer. Their unique electronic, optical and layer-polarized properties, and room-temperature stability will be easily incorporated through making heterostructures with various 2D materials, constructing new functional devices for practical use [49,50,51,52].

### 3.3. Transition Metal Carbides and Nitrides (MXene)

**Figure 5:**
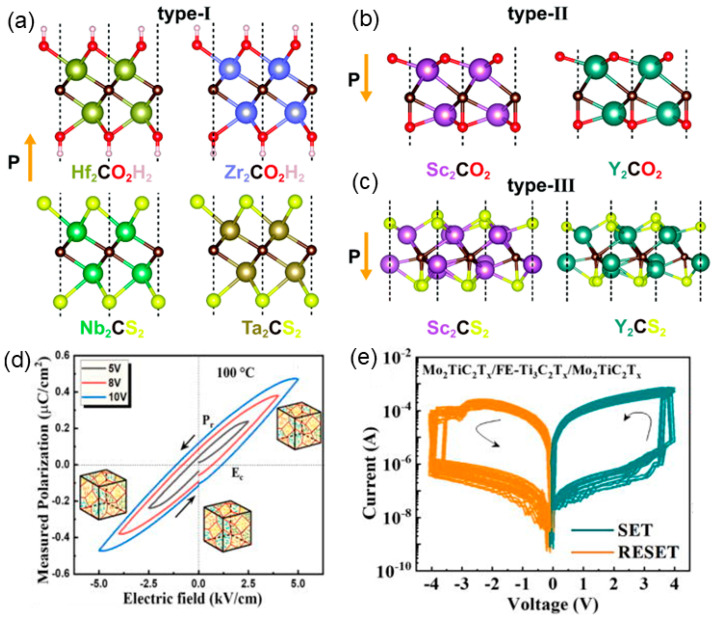
(**a**) The geometries of stable type−I (**a**), II (**b**), and III (**c**) FE MXenes [53]. (**d**) FE hysteresis loops of the Ti_3_C_2_T_x_ MXene film at 100 °C [54]. (**e**) Nonvolatile bipolar switching in single cell for logarithmic I–V measurements [54]. Reprinted with permission from [53], 2020, Royal Society of Chemistry; and [54], 2023, AIP Publishing.

A family of 2D materials, the monolayer MXene (M denotes the early transition metal, and X is carbon or nitrogen), have been synthesized successfully, attracting great attention for their applications in energy, thermoelectric, and optoelectronic fields [53,54,55,56]. Their bare surface with transition metal atoms exhibits high activity, where suitable functionalization can induce ferroelectricity. Among 2D FE materials discovered to date, which should possess the broken lateral centrosymmetry, those with out-of-plane polarization are limited. Compared with common approaches in the realization of out-of-plane polarization through halogen intercalation and vdW stacking, surface functionalization is more convenient to control in experiments. The oxygen-functionalized MXene Sc_2_CO_2_ monolayer was predicted to show in-plane and out-of-plane ferroelectricity, and its vertical polarization reached 1.60 μC/cm^2^ [57], almost an order of magnitude higher than that of 1T-MoS_2_ [41]. The vertical polarization mainly originates from the charge density overlap between O and C atoms, leading to the asymmetrical displacement that the negatively charged C atoms stay away from positively charged Sc layers. According to a multistep process of O atoms shifting, the transformation of polarization reversal undergoes an AFE intermediate state, and a energy barrier equal to or lower than 0.52 eV/f.u. is overcome, which is comparable with that of traditional perovskite FE LiNbO_3_. Interestingly, functional group modifications may not always induce ferroelectricity directly. For example, fluorine-functionalized Mxene Hf_2_CF_2_ is nonpolar. Under a moderate compressive strain, it transforms to a polar state as the F-induced symmetry is broken, generating the vertical ferroelectricity. An energy barrier of ~410 meV/f.u. is suggested to be the upper limit of the vertical ferroelectricity that can be generated in the systems [58]. A class of experimentally adopted functional groups are utilized to decorate MXenes, realizing ferroelectricity. Using high-throughput search DFT calculations, Zhang et al. performed a screening of 110 MXene candidates modified by OH, O, S, F, and Cl [53]. They identified three types of stable FE MXene phases, i.e., type-I: Nb_2_CS_2_ and Ta_2_CS_2_; type-II: Sc_2_CO_2_ and Y_2_CO_2_; and type-III: Sc_2_CS_2_ and Y_2_CS_2_, as shown in Figure 5a–c [53]. The remarkable in-plane and out-of-plane spontaneous polarization (4.93~6.25 × 10^−0^ C/m and 0.03~0.1 × 10^−10^ C/m, respectively) renders them with 2D ferroelectricity for their application in next-generation memories. It is found that most of them are easier to realize polarization reversal with a lower energy barrier (0.52~0.57 eV/f.u.) than that of FE vacancy-induced CrI_3_ (0.65 eV), mainly because the displacement of the functional groups facilitates polarization reversal. The results show the feasibility of creating FE MXene by chemical modification on its surface. The Nb_2_NF_2_ monolayer is another kind of MXene that is predicted to exhibit vertical polarization (0.046 eÅ) [59]. Notably, it belongs to the rare category of materials known as “FE metals”, vastly assisting the discovery of new 2D FE materials [59]. For the Sc_2_CO_2_ monolayer, FE ground states are predicted, in contrast to the zero polarization predicted in previous work [53]. For the multiferroic Hf_2_VC_2_F_2_ monolayer, a type of MXene containing double transition metal elements, the ferroelectricity is found to originate directly from its magnetism [60]. Specifically, the noncollinear 120° Y-type spin order breaks inversion symmetry, generating a polarization perpendicular to the spin helical plane. Although the polarization (1.98 × 10^−6^ μC/m) of the Hf_2_VC_2_F_2_ monolayer is much smaller than those of other 2D FE materials, the mechanisms of their ferroelectricity are conceptually different.

MXenes are promising candidates for data storage applications, such as nonvolatile memory. Fatima et al. created all-MXene FE random access memories based on a “Mo_2_TiC_2_T_x_/FE-Ti_3_C_2_T_x_/Mo_2_TiC_2_T_x_” trilayer scheme [54]. After being heat-treated, the Ti_3_C_2_T_x_ film transformed from non-FE to FE states with hysteresis loops, as shown in Figure 5d. The electrical I-V behavior indicated its nonvolatile bipolar switching with a similar on/off ratio in systems with 1–6 cells. The shift between high resistance state and low resistance state under various voltages is described in Figure 5e, demonstrating good reproducibility and endurance.

### 3.4. Group-IV Monochalcogenides

**Figure 6:**
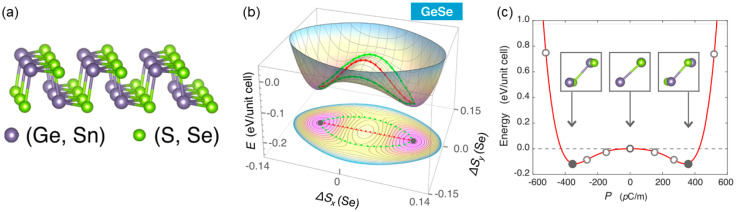
(**a**) Perspective view of GeSe monolayer [63]. (**b**) Potential energy surface with fractional shift of Se atoms and the corresponding contour plots [63]. (**c**) Double-well potential of GeSe monolayer and the atomic configurations with specific polarization [63]. Reprinted with permission from [63], 2017, IOP Publishing.

Similar to phosphorene, 2D group-IV monochalcogenides MX (M = Ge, Sn; X = S, Se, Te) possess hinge-like configurations, showing great potential in FE applications. The spontaneous in-plane electrical polarization and ferroelectricity induced by the unique ionic-potential anharmonicity in monolayer MX were predicted and analyzed [61]. Their special structure, stacked alternatively by M and X atoms with different electronegativity, leads to a relative in-plane displacement and thus results in robust room-temperature ferroelectricity, as shown in Figure 6a [62]. By searching the whole potential energy surface, two FE ground states are located at the two lowest points of the potential energy well, as shown in Figure 6b. The relationship between the total energy and spontaneous polarization also presents the characteristics of double well potential, as shown in Figure 6c. The total polarization calculated using the Berry phase theory is as high as 484 pC/m, 357 pC/m, 260 pC/m, and 181 pC/m for GeS, GeSe, SnS, and SnSe monolayers, respectively [63]. Considering the fact that an FE transition is governed by 180° domain wall motion, the remarkably small energy barrier for the GeS monolayer (~1.6 meV Å^−1^) suggests that its ferroelectricity can be verified by experimental observation and device characterization.

The GeTe monolayer with a stable orthorhombic phase was also reported to possess in-plane ferroelectricity above room temperature (*T*_c_ = 570 K) [64]. Under applied tensile strains, *T*_c_ could be improved significantly. Guan et al. reported the spontaneous in-plane polarization in boat-shape monolayer β-GeSe, which possesses a polarization comparable with that of the SnTe monolayer [65]. The β-GeS monolayer and the few-layer β-GeS were also predicted to possess in-plane ferroelectricity, with a spontaneous polarization of 2.00 × 10^−10^ C/m [66], while in the δ-GeS monolayer, the noncollinear ferroelectricity combining the FE mode along the *x*-axis and the AFE mode along the *y*-axis was studied [67]. The molecular dynamics simulation reveals a distinct phase transition around 500 K, indicating that the δ-GeS monolayer is promising for device applications at room-temperature.

**Figure 7:**
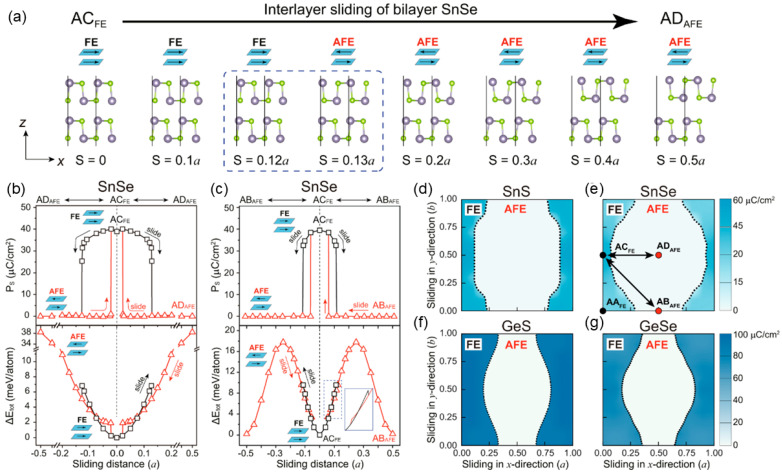
(**a**) Description of sliding processes. FE polarization and total energy difference as functions of the sliding distance for bilayer SnSe shifted (**b**) from AC to AD and (**c**) from AC to AB stacking sequences. (**d**–**g**) Contour plots of spontaneous polarization as a function of mechanical sliding distance for SnS, SnSe, GeS, and GeSe, respectively [68]. Reprinted with permission from [68], 2022, Springer Nature.

In view of the distorted and wrinkled structure of the MX monolayer, Xu et al. investigated the intralayer polarization reversal by mechanical interlayer sliding (tribology) [68]. They studied four stacking modes in bilayer MX, including those with FE and AFE states. Figure 7a shows the sliding pathway for the SnSe monolayer. As the top layer moves along the x direction, the horizontal Sn-Se bonds break, and the vertical Sn-Se bonds change their orientation, leading to FE-to-AFE transitions. The polarization and energy as a function of sliding distance with different pathways are shown in Figure 7b,c. Specially, the polarization drops to zero across the phase boundary, as shown in Figure 7d–f. Its ultrahigh rate of polarization change endows MX with high electrical performance when used in nanogenerators [68].

Besides the aforementioned theoretical predictions, there are experimental demonstrations of the ferroelectricity in 2D group-IV monochalcogenides. In 2016, Chang et al. discovered the in-plane polarization in SnTe film with a thickness at the 1 unit cell limit, as prepared by the molecular beam epitaxial technique. Its *T*_c_ reaches 270 K, much higher than that of its bulk counterpart (about 100 K) [18]. The theoretical studies carried out by Liu et al. revealed that the switching barrier and *T*_c_ in freestanding and defect-free SnTe films increased with increasing thickness if the thickness was less than 5 unit cells [69]. Higher *T*_c_ and polarization were further found in the few-atomic thick SnTe films with a orthorhombic phase [70], featuring antipolar inter-layer coupling and underlining their potential for the development of novel spontaneous polarization-based devices. For few-layer SnS showing purely in-plane ferroelectricity, as validated in experiments, an odd–even effect on the ferroelectricity was discovered, i.e., only an odd number of layers broke the centrosymmetry of SnS, which highlighted the possibility of controlling the ferroelectricity in multilayer structures [71].

### 3.5. Indium Selenide

**Figure 8:**
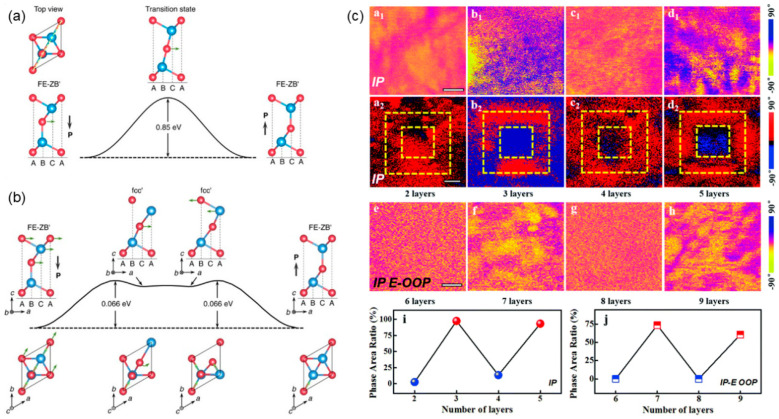
The energy barrier of polarization reversal in α-In_2_Se_3_ through (**a**) one-step and (**b**) three-step concerted mechanisms [72]; In and Se atoms are in blue and red, respectively. (**c**) In-plane (initial a_1_–d_1_ and switched a_2_–d_2_ states) and out-of-plane (switched e–h states) PFM phase images of 2H-stacked α-In_2_Se_3_ with different layers [73]. Reprinted with permission from [72], 2017, Springer Nature; and [73], 2021, Royal Society of Chemistry.

The group III-VI semiconductors crystallized with a layer structure are an important class of 2D materials, which have been extensively studied. Indium selenide (In_2_Se_3_), one of the most popular group III-VI compounds, consists of quintuple layers (Se-In-Se-In-Se) with weak vdW interaction. In 2017, based on DFT calculations, α-In_2_Se_3_ monolayers were identified to be FE with both in-plane and out-of-plane spontaneous polarization at room temperature [72]. The vertical polarization comes from the difference in the distances between the Se layer and two In layers, which leads to unequal In-Se bonds. Thus, the polarization can be reversed by a one-step (shifting the central Se layer laterally) or three-step concerted mechanism (transforming from a nonpolar phase), as shown in Figure 8a,b [72]. The mechanisms revealed are groundbreaking in predicting ferroelectricity in 2D materials.

The ferroelectricity of 2D In_2_Se_3_ was experimentally verified by Zhou et al [16]. There were two areas with the opposite phase contrast of 180° in PFM phase and amplitude images, indicating the upward and downward polarization. The out-of-plane polarization could be maintained until the thickness was down to ~10 nm. Further investigations indicated that such polarization was stable with a lower thickness (2~6 nm) [74,75,76]. Even if the number of nanoflakes was reduced down to a bilayer or monolayer, the room-temperature state still persisted [77]. Interestingly, the direction of polarization showed a dependence on the number of layers [74]. There was a difference of 180° in the PFM phases for the odd-layer and even-layer, unveiling the opposite direction of polarization in alternating layers. Such layer-related ferroelectricity was also confirmed in 2H-stacked In_2_Se_3_ [73], where the odd-layer dipole was nonzero and the even-layer dipole was zero, as shown in Figure 8c. Furthermore, It was found that there existed a locking between the out-of-plane dipoles and in-plane lattice asymmetry, which could be a new mechanism, that an out-of-plane FE state was stable against the depolarization field in the atomic-thick films [78]. Zhao et al. elaborated the mechanism for the existence of out-of-plane polarization in an In_2_Se_3_ monolayer, further suggesting that external stimuli (such as applied strains) could lead to a polar ground state in 2D M_2_X_3_ (M = In, Bi; X = Se, Te) monolayers with robust out-of-plane polarization [79].

Due to its strong out-of-plane polarization and semiconducting properties, α-In_2_Se_3_ is an ideal candidate for applications in nonvolatile memories. Wan et al. investigated a 2D FE FET composed of ultrathin α-In_2_Se_3_ and graphene, which demonstrated a robust nonvolatile performance where the random write cycles were more than 10^5^ [80]. The study provided a prototype for rewritable memory based on 2D vdW materials and highlighted their application for high-density information storage. Additionally, α-In_2_Se_3_ could also store and retrieve optical information, making it suitable for optoelectronic memory applications [81]. The planar memristor, utilizing its in-plane polarization, exhibited a noticeable switchable photocurrent and FE polarization; the devices based on α-In_2_Se_3_ with out-of-plane polarization could allow for high-density integration (7.1 × 10^9^ inch^−2^) and a high resistance-switching ratio (over 10^3^). As described above, the theoretically and experimentally tuned functionality in 2D In_2_Se_3_ are related to the absence of dangling bonds in the vdW heterostructures.

### 3.6. Other 2D FE Materials

In addition to the main 2D ferroelectrics that have been synthesized successfully, there are plenty of promising candidates proposed in theoretical calculations to guide experiment observations. For example, mono-elemental ferroelectricity was designed in multilayer graphene with the layer number N > 3 [82]. Different from the sliding ferroelectricity in bilayer stacking, the polarization in such 2D system is caused by the symmetry breaking related to the across layers instead of adjacent layers. The moiré pattern can also be observed with nonzero polarization in the twisted multilayer rather than in the bilayer. Tellurium is a candidate of 2D FE materials that has a layered structure similar to that of MoS_2_. The β-Te bilayer was theoretically predicted to have a structural distortion with a spontaneous in-plane polarization (1.02 pC/m) [83], while the α-Te bilayer was reported to exhibit out-of-plane polarization (0.49 pC/m) generated by asymmetric stacking [84]. In addition, robust ferroelectricity of the α-SbN monolayer was discovered in theoretical work, which had a very large in-plane spontaneous polarization of about 7.81 × 10^−10^ C/m. The polarization can be further modulated by applying strains on the monolayer [85]. In other Sb-containing 2D materials, single-layer γ-SbP and γ-SbAs maintain their ferroelectricity, with a polarization of 3.80 × 10^−10^ C/m and 3.47 × 10^−10^ C/m above room temperature, respectively. Similar to the nonlinear magnetism in magnetic monolayers, nonlinear ferrielectricity could stem from the competition between FE and AFE soft modes in 2D FE systems. It was found that 2D dioxydihalides MO_2_X_2_ (M: group-VI transition metal; X: halogen) exhibited nonlinear ferrielectricity, manifesting itself with unique features of polar structures (such as atomic-scale vortices in the domains) [86]. In contrast, monolayer VOX_2_ (X = Cl, Br, and I) was predicted to possess a large polarization [87]. Ding et al. investigated the ferrielectricity of monolayer VOI_2_ and pointed out that the effective Dzyaloshinskii–Moriya interaction in the polar monolayer resulted in the FE insulating state or ferromagnetic metallic state rather than the ferromagnetic FE states [88]. In a theoretical calculation on monolayer NbOX_2_ (X = Cl, Br, I), the coexistence of in-plane ferroelectricity and antiferroelectricity was revealed, while the latter was found to effectively reduce the energy barrier and thus promoted polarization switching.

The low-symmetric multiferroic ReWCl_6_ monolayer has two inequivalent phases, which exhibit polarization in opposite directions. Similar to that of FE perovskite oxides, the polarization (~27 µC/cm^2^) is caused by the displacement of cations in anion octahedral cages [89]. Particularly, an applied electric field simultaneously manipulates phase transition and in-plane polarization reversal in the monolayer, inducing special magnetoelectric coupling effects that can be found in similar systems [90]. Ferroelectricity was also found in single-layer FeO_2_H due to its broken mirror symmetry, which showed a spontaneous polarization of 0.68 × 10^−10^ C m^−1^ and a switching barrier of 0.457 eV/unit cell [91]. The layered β-ZrI_2_ material, which was a semiconducting counterpart of the isostructural semimetals such as MoTe_2_ and WTe_2_, was predicted to possess vertical ferroelectricity [92]. Ma et al. revealed that the interaction between the sliding displacements and charge redistributions in trilayer ZrI_2_ could be responsible for the ferroelectricity [92]. Its stable FE domain wall and low switching barrier could provide insights for further studies on “slidetronics”. Ding et al. analyzed the origin of polar ZrI_2_ from a structural perspective and estimated the magnitude of out-of-plane polarization (0.39 μC/cm^2^), which could be modulated by applied strains and hydrostatic pressure. Beyond the existing paradigm for ferroelectricity, Zhang et al. reported that bilayer ZrI_2_ achieved six-logic-state multiferroicity, which was characterized by the existence of crystal-symmetry-related 120° ferroelasticity and interlayer-sliding-induced in-plane and out-of-plane ferroelectricity. The above-mentioned theoretical studies put forward a novel group of 2D FE systems for applications in multifunctional nanodevices, which are awaiting experimental verification.

Moreover, ferroelectricity can be realized through manipulating 2D systems. For example, due to the large buckling in the h-NbN monolayer, its polarization is not readily switchable with an applied electric field while accessible in the monolayer under applied strains (4.85~5.3%), demonstrating strong coupling of electronic and photonic ferroelectricity [93]. In nonpolar bilayer PbXs (X = S, Se, Te), the lattice distortion occurs under applied strains, and the off-center displacement of Pb activates the in-plane polarization [94]. Ferroelectricity can be also achieved in 2D multilayers stacking with different orders. Since the spatial inversion symmetry is broken in 3R-type bilayer BX (X = P, As, Sb), spontaneous vertical polarization is induced, which can be switched through specific interlayer sliding [95].

## 4. Conclusions and Outlook

Recently, high-performance ferroelectrics have been in great demand for next-generation nano-electronic devices where multi-functionalization and miniaturization are required. It is currently an urgent task that novel low-dimensional ferroelectrics such as nano-array, nanoribbon, and 2D FE materials can be explored and designed for practical use, which remain challenging due to their disadvantageous FE properties. First is the disappearance of ferroelectricity due to depolarization in low-dimensional materials; second, there exists instability in ferroelectricity at room temperature because of the low Curie temperature of low-dimensional ferroelectrics; third, low-dimensional ferroelectrics possess a high energy barrier for polarization reversal, which hinders the implementation of switchable polarization for device applications. With the rapid development of 2D materials, 2D FE materials have become promising candidates for nano-electronic applications. However, existing 2D FE materials are scarce, and the aforementioned issues have not been well resolved. Computational study has played an important role in developing 2D FE materials. Further theoretical predictions for 2D FE materials should focus on several key areas.

First, accurate and efficient DFT calculations are beneficial in discovering 2D ferroelectrics with high performance and providing theoretical guidance for experimental verification. For example, the sliding ferroelectricity model for vdW bilayers, firstly proposed in 2017, offers a new prospective on materials design [44]. Only three years later, several research groups experimentally verified the model for a BN bilayer, measuring polarization that was consistent with theoretical predictions [51,52]. Computational approaches allow for efficient screening of properties, exploration on phase stability, and identification of favorable FE characteristics before experimental synthesis. However, accurate modeling of strain effects, defects, and electron–phonon interactions remains challenging for 2D FE materials. Tackling these complexities could facilitate our understanding of how atomic and electronic structures influence ferroelectricity and polarization dynamics of 2D FE materials.

Second, an in-depth understanding of the mechanisms of ferroelectricity, including those related to structural transformation, electronic structures, magnetism, and external stimuli, is critical. Such understanding enables precise design and screening of high-performance 2D FE materials. For example, by examining the polarization stability and response under applied strains or modulation on the number of layers, the FE properties can be accurately tuned.

Third, it is an effective strategy that 2D FE materials are combined with other 2D materials to form heterostructures, optimizing device performance. For instance, constructing heterostructures by stacking FE layers with 2D materials such as graphene, TMD, or topological insulators can enhance functional properties and broaden device versatility. By fine-tuning interfacial coupling effects, those heterostructures enable controlled modulation of charge, spin, and polarization, which hold promise for applications in memory storage, logic devices, and sensors. Due to their strong compatibility with fabrication processes in the modern electronics industry, 2D FE materials can be seamlessly integrated to create multifunctional devices. Precise manipulation and optimization on layer stacking of 2D FE materials could lead to low-cost, high-performance nano-electronic devices with enhanced stability and durability.

From an application perspective, besides their nano-electronic applications, 2D FE materials are also promising for nano-photonic applications. Two-dimensional FE materials with inherent noncentrosymmetry are prime candidates for second harmonic generation (SHG), a fundamental nonlinear optical process typically generated in noncentrosymmetric materials. In the SHG process, two photons of frequency ω are combined to yield a single photon of frequency 2ω within a material. The layered FE niobium oxide dihalides NbOX_2_ (X = Cl, Br, and I) are found to exhibit a layer-independent SHG response, distinct from other known nonlinear 2D materials [96,97]. Their anisotropic structure leads to an anisotropic band structure and optical response, which can be modulated to control light–material interactions. For example, the SHG intensity can be effectively modulated by applied strains. In the NbOI_2_ monolayer, SHG strength doubles by bending the substrate due to the enhanced piezoelectric fields and distortions [98]. With a small strain (3.1%) along the polar direction, the SHG conversion efficiency of NbOI_2_ can increase by 35-fold [97]. Additionally, the SHG coefficient for the NbOCl_2_ monolayer can be significantly modified by strain-induced phase transitions [99].

Temperature and electric field also offer tuning capabilities for SHG signals generated in 2D FE materials. For instance, increasing the in-plane electric field can modulate SHG intensity by more than 80%, aligning with the polarization changes between paraelectric and FE states [97]. The tunability of spontaneous polarization of 2D FE materials, influenced by external stimuli such as electric fields, stresses, or temperature changes allows for dynamic control over SHG efficiency. Combined with their high SHG output and low-loss characteristics, layered FE materials show promising features for optoelectronics, data storage, sensing, and laser applications.

Despite the significant advances of 2D FE materials in nonlinear optical devices, future research should focus on improving SHG efficiency and further exploring underlying mechanisms. Moreover, integrating 2D FE materials with other platforms to maintain SHG stability remains a challenge. An effective method can be implemented by constructing heterojunctions. For example, in heterojunctions of air/indium tin oxide (ITO)/silica quantum wells [100], an ultrahigh SHG intensity can be achieved due to the ITO layer’s asymmetry, caused by varying electron affinities in the surrounding environment.

Looking ahead, collaborative efforts between theoretical modeling and experimental studies are essential. Advanced computational methods, combined with high-throughput experimental techniques, can provide faster feedback loops that will refine material properties and accelerate discoveries of 2D FE materials. The future of 2D FE materials is thus not just in their direct applications but also in their transformative impact across multiple advanced technologies.
